# Clinical Effect of Electroacupuncture on Lung Injury Patients Caused by Severe Acute Pancreatitis

**DOI:** 10.1155/2017/3162851

**Published:** 2017-06-29

**Authors:** Li Li, Jianbo Yu, Rui Mu, Shuan Dong

**Affiliations:** ^1^Department of Anesthesiology, Tianjin Central Hospital of Gynecology Obstetrics, Tianjin 300100, China; ^2^Department of Anesthesiology, Tianjin Nankai Hospital, Tianjin 300100, China

## Abstract

This study aimed to investigate the effects of electroacupuncture at the Lieque, Chize, and Zusanli points in patients with lung injury caused by severe acute pancreatitis. Patients with acute respiratory distress syndrome (ARDS) induced by severe acute pancreatitis (SAP) were randomly divided into three groups based on the treatment: conventional therapy alone (group A), electroacupuncture of nonacupoints with conventional therapy (group B), and electroacupuncture at the Lieque (LU7), Chize (LU5), and Zusanli (ST36) points (group C) once a day for 5 days. Arterial blood samples were obtained for blood gas analysis before electroacupuncture (*T*1) and 3 (*T*2) and 5 (*T*3) days after electroacupuncture. The oxygenation index was significantly higher in all groups at *T*2 and *T*3 than that at *T*1, while the APACHE-II scores were decreased significantly. The expression of TNF-*α* was significantly decreased and the IL-10 was significantly increased in all groups at *T*3. The oxygenation index at *T*2 and *T*3 was significantly higher in group C than that in group B. Electroacupuncture at Lieque, Chize, and Zusanli can lessen the lung injury induced by SAP, and the mechanism may be related to the decreased TNF-*α* and increased IL-10 value. Clinical Registration Number is ChiCTR-ICR-15006850.

## 1. Introduction

Severe acute pancreatitis (SAP) is a common acute syndrome that is characterized by pancreatic digestion by its own enzymes and necrosis. SAP is difficult to treat with conservative measures and therefore requires surgery. It can often cause systemic inflammatory response syndrome (SIRS) [1] and the associated mortality rate ranges from 25 to 30% [2–6]. Related researches have illustrated that lung is the earliest and most easily damaged organ in SAP. Acute pancreatitis-associated lung injury (APALI) is the most common early complication, and acute lung injury is the main cause of high mortality in SAP [7]. Unfortunately, none of the currently available drugs are effective in treating SAP [8]. Therefore, determining how to effectively regulate the inflammatory reactions in SAP and prevent the consequent lung injury is of great clinical significance.

Within the concepts of traditional Chinese medicine (TCM), maintenance of health and normalization of body function stems from a balance of “yin” and “yang” and free flow of energy (Qi) along various meridians in the body. Previous studies have suggested that acupuncture, a technique which facilitates free flow of Qi, can alleviate dyspnoeic symptoms, reduce postexercise bronchoconstriction, and improve exercise capacity. Studies have demonstrated that electroacupuncture can regulate the release of inflammatory factors and immune stress response [9]. Electroacupuncture at the Zusanli (ST36) point in rats has been shown to inhibit the release of inflammatory mediators and reduce endotoxin-induced acute kidney injury [10]. Electroacupuncture at the Zusanli point has a bidirectional benign regulatory role in the pituitary-adrenal cortex system function and improves the immune ability [11]. The Lieque (LU7) point is an important acupuncture point on the lung meridian, which is clinically used in the treatment of respiratory diseases. Studies have shown that electroacupuncture at the Lieque point could improve the symptoms and pulmonary functions of patients with asthma or chronic obstructive pulmonary disease by regulating the autonomic nervous system, promoting the release of endogenous peptide at the hypothalamic level, and inhibiting the release of inflammatory cytokines [12–14]. Chize (LU5) belongs to the lung meridian, which also participates in the regulation of lung functions according to the theory of traditional Chinese medicine (TCM). Pairing of the viscera and bowels is an important theory, which provides guidance to TCM clinical practice. Recently, researchers have performed many studies on the theory that the lung and large intestine are exterior-interiorly related, which is a different point of view to that of previous literature; recent clinical studies and experimental studies have enforced the theoretical connotation of the statement. Studies have also shown that electroacupuncture stimulation at Chize could effectively inhibit the effect of enteritis on the lungs, decrease the level of tumor necrosis factor- (TNF-) *α*, and reduce the ulcerative colitis-induced lung injury significantly [15]. During clinical treatment, different acupuncture points that are compatible with medicines are selected for treating diseases. The present study intended to apply electroacupuncture at the Lieque, Chize, and Zusanli points to determine whether this treatment could improve the degree of lung injury caused by SAP.

## 2. Materials and Methods

**Figure 1:**
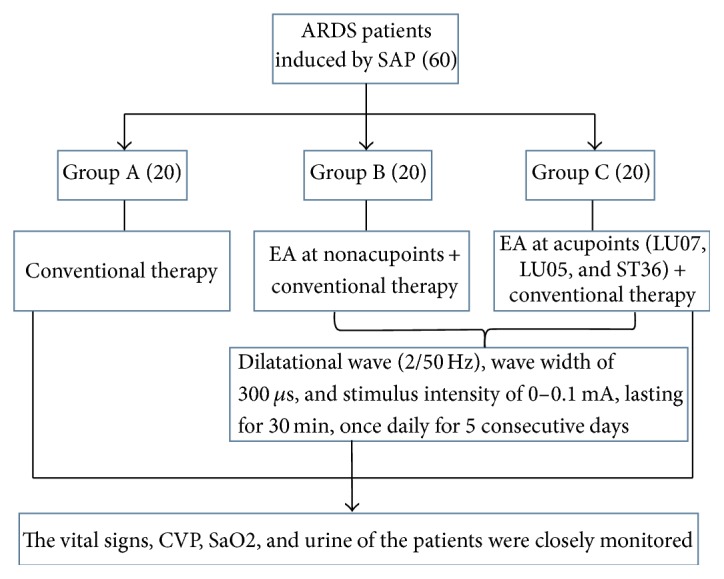
Flowchart about the group.

**Figure 2:**
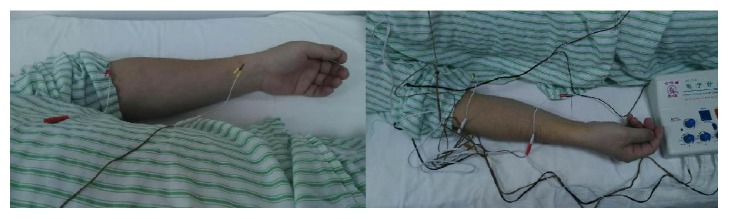
Electroacupuncture was treated at Lieque (LU7) and Chize (LU5).

**Figure 3:**
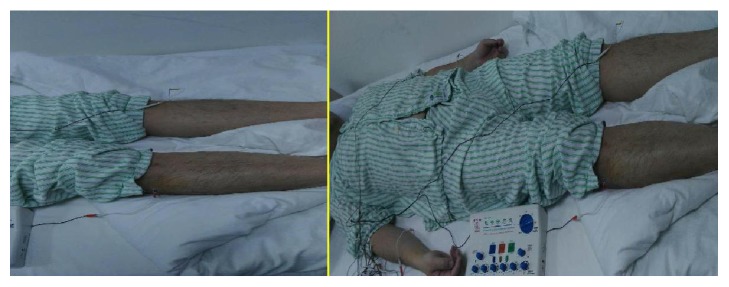
Electroacupuncture was treated at Zusanli (ST36).

**Figure 4:**
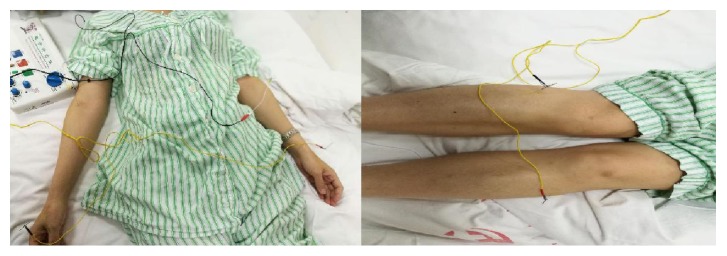
Electroacupuncture of nonacupoints (one centimeter away from Lieque, Chize, and Zusanli points but different from the traditional Chinese medicine “Jing Luo”).

The present research was approved by the Medical Ethics Committee of Nankai Hospital, Tianjin City (number tjnk20130618), Clinical Registration Number: ChiCTR-ICR-15006850. Informed consent was obtained from the patients and/or their families. Total of 60 patients who were newly diagnosed as acute respiratory distress syndrome (ARDS) induced by SAP were recruited. Exclusion criteria included previous bronchial and pulmonary diseases, recent hormone use, recent immunosuppressive therapy, and recent acupuncture treatment. ARDS was diagnosed according to the ARDS diagnostic criteria chaired and revised by the European critical disease association in Berlin of Germany in 2011 [16]. Patients were randomly divided into three groups (*n* = 20) using a computer-generated random number table. All the acupuncture treatments were completed by the same physician with 10 years of clinical experience and professional operational skills. In group A, only conventional therapy was conducted without acupuncture treatment. In group B, electroacupuncture of nonacupoints (one centimeter away from Lieque, Chize, and Zusanli points but different from the traditional Chinese medicine “Jing Luo”) was conducted based on conventional therapy with the same acupuncture parameters as those in group C. In group C, electroacupuncture was carried out at the Lieque point (LU7; on the radial aspect of the forearm, between the tendons of the abductor pollicis longus and the extensor pollicis brevis muscles, in the groove for the abductor pollicis longus tendon, and 1.5 B-cun superior to the palmar wrist crease), Chize point (LU5; on the anterior aspect of the elbow, at the cubital crease, and in the depression lateral to the biceps brachii tendon), and Zusanli point (ST36; on the anterior aspect of the leg, on the line connecting ST35 with ST41, and 3 B-cun inferior to ST35) based on the conventional therapy (Figures 1–4). Dilatational wave (2/50 Hz) was selected with a wave width of 300 *μ*s. The stimulus intensity was gradually increased from 0 with a gradient of 0.1 mA, which caused sour, hemp, swelling, and heavy feelings, suggesting that “acupuncture brings about the desired sensation.” [17] Then, the stimulus intensity was maintained at this level for continuous stimulation for 30 min, and the treatment was administered once daily for 5 consecutive days. The treatment criteria of the three groups were based on the diagnosis and treatment guidelines for acute lung injury/ARDS. The vital signs, CVP, SaO2, and urine of the patients were closely monitored [18, 19].

### 2.1. Data Collection

All the patients were included in the study after they were confirmed as having APALI in 24 h. Arterial blood samples were drawn and arterial blood gas analysis indices were detected before (*T*1) and 3 (*T*2) and 5 (*T*3) days after electroacupuncture treatment using a GEM3000 blood gas analyzer for all three groups. The oxygenation index (PaO2/FiO2) was calculated for determining the APACHE-II scores. APACHE-II (“Acute Physiology and Chronic Health Evaluation II”) is a severity-of-disease classification system, one of several ICU scoring systems. It is applied with 24 hours of admission of a patient to an intensive care unit (ICU): an integer score from 0 to 71 is computed based on several measurements; high scores correspond to more severe disease and a higher risk of death. Then, 5 mL of peripheral blood samples was collected from all three groups at *T*1 and *T*3. The blood samples were allowed to rest at room temperature for 30 min or at 4°C overnight, after which they were centrifuged at 2000 rpm/min for 20 min to obtain sera, which were collected and preserved at 20°C. Fiber bronchoscopy and alveolar lavage were used to collect samples. Approximately 5–10 mL of bronchoalveolar lavage fluid (BALF) was collected. After centrifugation at 2000 rpm/min for 20 min, the supernatant was collected and preserved at 20°C, in order to avoid repeated freezing and thawing. An ELISA kit, purchased from Wuhan Youersheng (USCN) Technology Co., Ltd., was used to determine the TNF-*α* and IL-10 concentrations in the BALF and serum, according to the manufacturer's protocol. The primary observation indices were employed to calculate the oxygenation index (PaO_2_/FiO_2_). The secondary observation indices included the TNF-*α* and IL-10 concentrations in the serum and BALF. The acute pathophysiology and chronic health assessment- (APACHE-) II score was used in the present study.

### 2.2. Statistical Analysis

SPSS 18.0 was used for statistical data analysis of the data obtained in this study. Data are shown as the number of subjects, mean ± standard deviation, or mean ± standard deviation of the mean. Comparisons between groups were performed using the *χ*^2^ test for categorical variables. The continuous response variables like age, weight, PaO2/FiO2, and APACHE-II score are presented as mean ± SD. One-way ANOVA was applied to compare the means among the 3 groups. Variables with repeated measures, such as arterial blood gas analysis parameters, were analyzed using repeated measures analysis of variance. *P* < 0.05 was regarded as a significant difference.

## 3. Results

There were no significant differences in the gender constitution, age, body weight, and APACHE-II scores before treatment and the oxygenation index between the three groups showed also no significant difference (*P* > 0.05; Table 1). The oxygenation index in all groups at *T*2 and *T*3 was significantly improved compared with that at *T*1 and APACHE-II scores were decreased (*P* < 0.05; Tables 3 and 4). Compared with group A, the oxygenation index of group C increased at *T*2 and *T*3, and the APACHE-II score decreased at *T*3 (*P* < 0.05; Table 3). In comparison, there was no significant difference in the above parameters in group B (*P* > 0.05; Table 2). The serum and BALF concentrations of TNF-*α* in the three groups decreased at *T*3 compared to *T*1, while the concentration of IL-10 increased (*P* < 0.05; Tables 3 and 4). Compared with the values in group A, the serum and BALF concentrations of TNF-*α* in group C decreased at *T*3 while the concentration of IL-10 increased (*P* < 0.05; Table 3). In comparison, there was no significant difference between the above parameters in group B (*P* > 0.05; Table 2). There were no significant differences in the side effects between the three groups (*P* > 0.05; Table 5).

## 4. Discussion

TNF-*α* is a cytokine that is derived from a number of cells, but its main sources are the macrophages and monocytes. It interacts with a number of other cytokines such as IL-1, IL-6, and platelet activation factor (PAF) and has a pivotal role in the inflammatory response. It has a short plasma half-life, of 14–18 min, due to rapid clearance by the liver, gastrointestinal tract, and kidney, making its presence difficult to assess by serum assays. Therefore, absent or low levels of TNF in serum do not correlate well with actual events in the internal milieu. While it is difficult to measure TNF levels in peripheral blood, certain studies have shown increased TNF levels in 30%–40% of patients with AP. In experimental studies, IL-10 has been shown to decrease levels of inflammatory markers and reduce the severity of pancreatitis. In experimentally induced pancreatitis, IL-10 levels parallel serum TNF levels and anti-IL-10 treatment reduce lung injury and pancreatic acinar necrosis, as well as reducing mortality from 42% to 0%. Synthetic IL-10 agonist pretreatment reduced lung injury and mortality from experimental pancreatitis. The results of the present research illustrate that the level of the proinflammatory factor TNF-*α* decreased while the level of the anti-inflammatory cytokine IL-10 increased in patients with SAP after they received conventional therapy in combination with electroacupuncture at the Zusanli, Lieque, and Chize points, indicating that electroacupuncture at the three acupoints can flexibly promote the level of proinflammatory factors in patients with SAP and elevate the concentrations of anti-inflammatory factors, both of which are vital in the treatment of patients with SAP. Some scholars have found that preventive electroacupuncture at Zusanli could alleviate acute lung injury induced by lipopolysaccharide in rats, and the mechanism involved the inhibition of TNF-*α* transcription [20]. In addition, electroacupuncture at Zusanli exerted an anti-inflammatory effect via the autonomic nervous system, attenuated the lipopolysaccharide-induced systemic inflammatory response, and improved the survival rate of rats with endotoxemia [21]. Both the Lieque and Chize points are important acupoints on the lung meridian, improving and treating respiratory system diseases and participating in the regulation of lung function. The electroacupuncture at the Lieque and Chize points through skin acupoints could regulate the autonomic nervous system, promote the release of endorphins in the hypothalamus, and inhibit the release of inflammatory factors and improve pulmonary function [12, 13]. A previous study has shown a negative correlation between APACHE-II scores and mortality of patients with ARDS [22]. The APACHE-II score is an important prognostic indicator for patients with ARDS. The results of the present research show that, in the patients with SAP who received conventional therapy combined with electroacupuncture at the Zusanli, Lieque, and Chize points, the oxygenation index was significantly higher, and the APACHE-II score was significantly decreased, suggesting that electroacupuncture at these three acupoints can effectively treat patients with acute lung injury [23]. The cause of death in patients with organ dysfunction is complicated and many interventions are required to treat these patients. In such cases, electroacupuncture can be used as an important measure to alleviate the lung injury induced by severe acute pancreatitis. The limitation of this study is its relatively small sample size. Because the current experiment is a single-center clinical trial, the number of patients who were admitted to this study is limited. Further research should aim to organize multicenter clinical studies based on the present study.

## 5. Conclusions

In summary, electroacupuncture at the Lieque, Chize, and Zusanli points can further improve the oxygenation index of patients with lung injury induced by SAP and alleviate the APALI, and the mechanism may be related to the downregulation of the TNF-*α* level and the upregulation of the IL-10 concentration.

## Figures and Tables

**Table 1:** Comparison of various general indicators in the three patient groups ($\overset{-}{x}\pm S$).

Indices	Group A	Group B	Group C	*P* value
Gender (cases, male/female)	11/9	12/8	11/9	0.934
Aged (years)	54 ± 12	58 ± 12	57 ± 11	0.5333
Body weight (kg)	67.5 ± 10.5	70.6 ± 9.6	62.6 ± 13.6	0.0894
PaO_2_/FiO_2_ (mmHg)	203.2 ± 31.4	215.2 ± 42.7	181.3 ± 58.4	0.0661
APACHE-II score	17.9 ± 3.3	20.2 ± 3.1	19.3 ± 3.7	0.1038

**Table 2:** Comparison of the parameters of groups A and B when there was a correlation between the groups ($\overset{-}{x}\pm S$).

	Group A	Group B	*P* value
Difference in the oxygenation index *T*_2_ − *T*_1_	16 ± 4	17 ± 6	0.2694
Difference in the oxygenation index *T*_3_ − *T*_1_	38 ± 3	36 ± 5	0.0667
Difference in score *T*_1_ − *T*_2_	1.4 ± 2.8	1.5 ± 1.2	0.4420
Difference in score *T*_1_ − *T*_3_	3.2 ± 1.5	3.8 ± 1.3	0.0922
Plasma TNF-*α* *T*_1_ − *T*_3_	3.4 ± 2.8	3.6 ± 1.9	0.3965
Plasma IL-10 *T*3 − *T*1	4.8 ± 2.9	5.1 ± 2.8	0.3705
BALF TNF-*α* *T*1 − *T*3	20.5 ± 5.3	22.3 ± 4.9	0.1359
BALF IL-10 *T*3 − *T*1	5.6 ± 4.2	6.1 ± 2.4	0.3233

**Table 3:** Comparison of the differences in parameters when there is correlation between groups A and C ($\overset{-}{x}\pm S$).

	Group A	Group C	*P* value
Difference in the oxygenation index *T*_2_ − *T*_1_	16 ± 4	48 ± 8	<0.0001
Difference in the oxygenation index *T*_3_ − *T*_1_	38 ± 3	85 ± 9	<0.0001
Difference in score *T*_1_ − *T*_2_	1.4 ± 2.8	2.8 ± 1.8	0.0338
Difference in score *T*_1_ − *T*_3_	3.2 ± 1.5	6.1 ± 1.2	<0.0001
Plasma TNF-*α* *T*_1_ − *T*_3_	3.4 ± 2.8	12.1 ± 3.5	<0.0001
Plasma IL-10 *T*_3_ − *T*_1_	4.8 ± 2.9	13.7 ± 3.8	<0.0001
BALF TNF-*α* *T*_1_ − *T*_3_	20.5 ± 5.3	25.6 ± 5.2	0.0020
BALF IL-10 *T*_3_ − *T*_1_	5.6 ± 4.2	22.8 ± 5.9	<0.0001

**Table 4:** Comparison of the differences in parameters when there is correlation between groups B and C ($\overset{-}{x}\pm S$).

	Group B	Group C	*P* value
Difference in the oxygenation index *T*_2_ − *T*_1_	17 ± 6	48 ± 8	<0.0001
Difference in the oxygenation index *T*_3_ − *T*_1_	36 ± 5	85 ± 9	<0.0001
Difference in score *T*_1_ − *T*_2_	1.5 ± 1.2	2.8 ± 1.8	0.0338
Difference in score *T*_1_ − *T*_3_	3.8 ± 1.3	6.1 ± 1.2	<0.0001
Plasma TNF-*α* *T*_1_ − *T*_3_	3.6 ± 1.9	12.1 ± 3.5	<0.0001
Plasma IL-10 *T*_3_ − *T*_1_	5.1 ± 2.8	13.7 ± 3.8	<0.0001
BALF TNF-*α* *T*_1_ − *T*_3_	22.3 ± 4.9	25.6 ± 5.2	0.0020
BALF IL-10 *T*_3_ − *T*_1_	6.1 ± 2.4	22.8 ± 5.9	<0.0001

**Table 5:** Comparison of the differences in side effects of three groups (%).

Group	Local pain	Local swollen	Local bleeding
A	2 (10%)	2 (10%)	1 (5%)
B	3 (15%)	3 (15%)	2 (10%)
C	2 (10%)	2 (10%)	1 (5%)
*P* value	0.851	0.851	0.765
